# Lindenshmidt’s Urethral Irrigators and Irrigating Dilators

**Published:** 1891-03

**Authors:** 


### LINDENSCHMIDT'S URETHRAL IRRIGATORS AND IRRIGATING DILATORS.

These instruments are among the best, if not the best instruments of this class ever invented.

They seem to fulfill every erequirement where an instrument of this kind is needed. Tyey are simple in construction, so that they are readily kept clean and aseptic, two great points in the treatment of diseases where their use is called for.

We can heartily recommend them from personal experience, for we have been using them for a long time in treating both disease of the bladder and urethra.

In irrigating the bladder we have found them particularly useful. For further information regarding them, write to the R. Hyde Co., Third and Prairie Sts., Milwaukee, Wis. A cut of them is given on our advertising pages.

